# Cystic Adventitial Disease of the (ilio) Femoral Artery with a Connection to the Hip Joint: Case Report and a Review of the Literature

**DOI:** 10.1016/j.ejvsvf.2022.01.014

**Published:** 2022-02-05

**Authors:** Misha R.M. Frenken, Carsten W.K.P. Arnoldussen, Roel J.L. Janssen

**Affiliations:** a Department of Vascular Surgery, VieCuri Medical Centre, Venlo, the Netherlands; b Department of Radiology, VieCuri Medical Centre, Venlo, the Netherlands

**Keywords:** Cystic adventitial disease, Femoral artery, Iliofemoral artery

## Abstract

**Objective:**

Cystic adventitial disease (CAD) is an uncommon non-atherosclerotic peripheral vessel disease, most often seen in the popliteal artery. Only a small number of cases involving the (ilio) femoral artery have been reported. The case of a 48 year old female with CAD of the left femoral artery with a connection of the disease to the hip joint on pre-operative imaging confirmed during surgery is described. A literature review of CAD of the (ilio) femoral artery with patient demographic data, symptoms, management, presence of a joint connection, and long term outcomes was performed.

**Methods:**

Multiple databases (Medline, CINAHL, EMBASE) were searched and each article was cross referenced to collect the literature on CAD of the (ilio) femoral artery. Case studies or series of CAD of the (ilio) femoral artery in English between 1995 and 2021 were included.

**Results:**

Sixteen case reports with 17 patients were included; 71% were male. CAD was unilateral in all case reports, with 53% on the right side. Patients presented with vascular symptoms including claudication (88%), a palpable pulsating mass (18%), acute limb ischaemia (6%) or limb swelling (8%). Computed tomography angiography (CTA) (76%) and duplex ultrasonography (47%) were the most commonly used imaging modalities. The common femoral artery was the most affected site (88%). Reported treatments were cyst resection and autologous vein reconstruction (six, one recurrence), cyst resection and patch repair (five, one recurrence), cyst resection with synthetic graft reconstruction (three, no recurrence), cyst resection (two, one recurrence), and cyst incision and decompression (one, one recurrence). In 18% of the cases, a connection between the CAD and hip joint was seen.

**Conclusion:**

Cyst resection and ligation with interposition of an autologous vein graft, synthetic graft or patch repair (in only locally affected arteries) seems to be the preferred treatment, with a low reported recurrence rate. CTA and magnetic resonance imaging are the imaging modalities of choice when suspecting CAD to determine an appropriate pre-operative plan and identify joint connections.

## Highlights

• CAD could be considered in relatively young patients with vascular symptoms.
• CTA and MRI should be the imaging modality of choice when suspecting CAD.
• Manage CAD with cyst resection and autologous/synthetic graft or patch interposition.
• Ligation of CAD is recommended to reduce the chance of recurrence.

## Introduction

Cystic adventitial disease (CAD) is a rare vascular disorder where mucinous cysts form within the adventitia of arteries and/or veins. It is thought that the outward pressure of the cysts (the cysts are “locked” within the adventitial layer(s) of the vessel) can cause a stenosis or occlusion of the vessel lumen resulting in vascular symptoms. It is more commonly described in arteries (90%) than veins, with the popliteal artery (80%) as the most affected site. It mainly affects middle aged men and can occur in the absence of atherosclerotic risk factors. Only a small number of cases involving the (ilio) femoral artery have been reported. There remains no consensus about the aetiology of CAD, with several hypothesis described in previous literature. The case of a 48 year old female with CAD of the left femoral artery with a connection of the disease to the hip joint on pre-operative imaging and confirmed during surgery is described. A literature review was performed of CAD of the (ilio) femoral artery with patient demographic data, symptoms, management, presence of a joint connection and long term outcomes.

## Case report

A 48 year old female was referred to the outpatient clinic with acute onset of intermittent claudication of her left leg. One year before, she presented with similar complaints which completely resolved after a short period. Further medical history was unremarkable. She was a non-smoker with a body mass index of 24.6, a normal blood pressure and low density lipoprotein values.

On physical examination, her peripheral pulsations on both sides were palpable but weaker on the left. Further vascular examination showed an ankle brachial index of 0.62 on the left and 1.17 on the right.

A contrast enhanced computed tomography (CT) scan of the pelvis and legs demonstrated a widening of the common femoral artery on the left side with a maximum diameter of 2.0 cm, also involving the origin of the superficial femoral and deep femoral arteries. The widening seemed to be almost completely occluding the arterial lumen. To confirm suspected CAD, additional contrast enhanced magnetic resonance angiography was done to provide more information.

**Figure 1:**
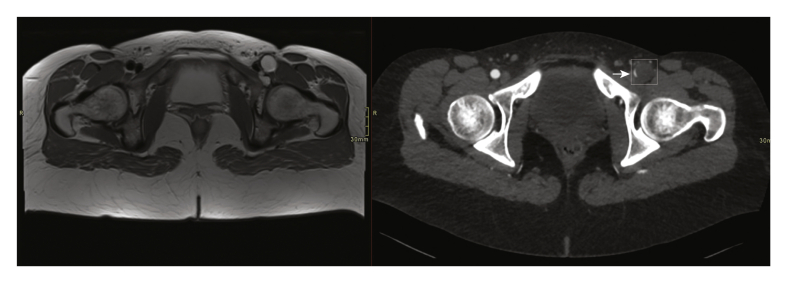
Images of the adventitial cyst at the level of the common femoral artery on the left (T2 weighted MRI). The image on the right shows a post-contrast CT angiogram with a slit like lumen of the common femoral artery (arrow) and a normal lumen on the right side of it. The hypodense dilated aspect of the common femoral artery on the left (box) is not a thrombosed dissection or aneurysm as we see on the T2 weighted MRI image that shows a bright (water) signal in the cystic structure.

**Figure 2:**
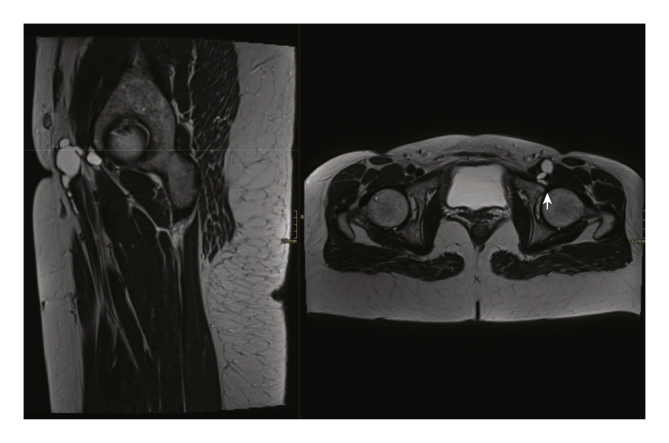
MRI images of the adventitial cyst in relation to the hip join on the left. T2 weighted images in the sagittal (left) and axial (right) plane showing the relationship between the cystic structure extending from the left hip joint (arrow) to the common femoral artery and proximal superficial femoral artery.

**Figure 3:**
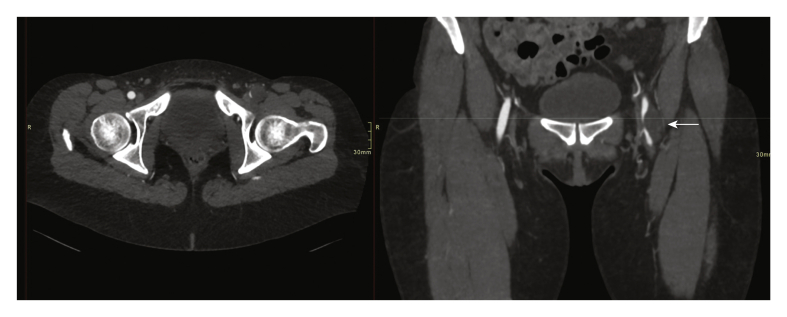
CT reconstructions in the axial (left) and coronal (right) plane at the level of the common femoral artery. The coronal image shows the slit like lumen of the common femoral vein in relation to the hypodense cyst (arrow).

This showed a lobulated ectatic cystic abnormality of the entire section of the common femoral artery which continued to the proximal superficial and deep femoral artery and resulted in a stenosis of approximately 75% of the vessel lumen. There was a connection between the cystic abnormality and the left hip joint (Figure 1, Figure 2, Figure 3).

**Figure 4:**
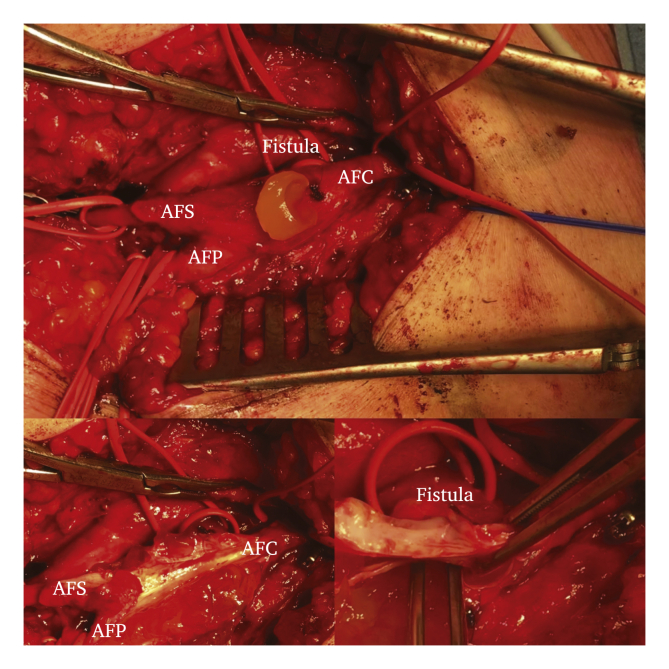
Peri-operative image with the affected common femoral artery with its bifurcation to the deep and superficial femoral arteries. A small incision is made in the adventitial layer with release of gelatinous material. Dissection of the adventitial layer with gelatinous material located between the layers of the tunica media and adventitia of the common femoral artery ending at the bifurcation to the deep and superficial femoral arteries. The vessel loop medially (located directly below the vascular clamp) is positioned around the section of the fistula canal as origin of the gelatinous material. The lumen of the artery is unaffected without sings of atherosclerosis or calcification. The fistula is explored with forceps and appears to have a connection to the left hip joint.

Intra-operatively, multilocular cystic lesions were found in the common, superficial, and deep femoral artery containing a gelatinous material. Vascular clamps were applied to the proximal common femoral artery and the proximal superficial and deep femoral arteries. An incision of the adventitia was made and the mucinous specimen was removed. The cystic abnormality was explored and revealed a connection to the anterior hip joint as seen on pre-operative imaging (Fig. 4). It was decided to carry out a complete resection of the femoral bifurcation and perform a reconstruction with the ipsilateral femoral vein as interposition graft instead of the great saphenous vein because of substantial diameter mismatch. The connection to the hip joint was ligated. Peripheral pulsations at ankle level were palpable post-operatively.

Histological examination showed a normal intima with an abnormal adventitia surrounded by cystic tissue, consisting of collagen with local accumulation of mucin and myxoid degeneration. The wall structure did not contain elastin, and only contained a few cells.

Duplex ultrasound at three and 12 months showed normal blood flow velocities in the venous interposition graft and absence of stenosis or recurrence.

## Methods

A systematic literature search was conducted across multiple scientific databases (Medline, CINAHL, EMBASE). Studies or case reports in English published between 1995 and 2021 that reported CAD of the (ilio) femoral artery were included. The following keywords were used: cystic adventitial disease, femoral artery. A total of 28 articles were found after the search. Sixteen case reports with 17 patients were included in this study. Reasons for exclusion were venous CAD, publishing date or language (non-English).

## Results

A total of 16 case reports with 17 patients were included.^1^, ^2^, ^3^, ^4^, ^5^, ^6^, ^7^, ^8^, ^9^, ^10^, ^11^, ^12^, ^13^, ^14^, ^15^ In Table 1, the patient demographic data are summarised. The mean age was 49 years. Twelve of 17 patients were male (71%). Eight of 17 patients had no medical history (47%), and four patients smoked (24%). In one case, trauma was reported as onset for the symptoms (6%) [10]. CAD was unilateral in all cases, with 53% on the right side. Claudication was the most reported symptom in 88% of the cases, one patient presented with acute limb ischaemia (6%). Three patients complained of a palpable mass in the groin (18%). Limb swelling was described in one case (6%) with a combined arterial and femoral vein involvement by CAD.^8^ CTA (76%) and duplex (47%) were the most used imaging modalities. In 10 cases, CAD was suspected or diagnosed pre-operatively (59%). In other cases, the diagnosis was made during surgery. All the cases were sent for histological evaluation to confirm the diagnosis of CAD. The common femoral artery is the most commonly affected site (88%). Cyst resection with autologous vein reconstruction was the most reported treatment (six, one recurrence). The other reported treatments were cyst resection with patch repair (five, one recurrence), cyst resection with synthetic graft reconstruction (three, no recurrence), cyst resection (two, one recurrence), and cyst incision and decompression (one, one recurrence). In three cases (18%), a joint connection (pre-operatively or during surgery) between the CAD and hip joint was seen (see Table 2, Table 3, Table 4, Table 5).^11^^,^^12^

**Table 1:** Patient demographics.

	Sex/Age (Years)	Comorbidities	Smoker	Trauma	Side	Symptoms
Maeda et al.^1^	Male/53	Diabetes, Gout	Unknown	No	L	Claudication progressive over three months
Park et al.^2^	Male/79	Hypertension, rheumatoid arthritis, COPD, CVA	Unknown	No	R	Calf claudication for one day
Gagnon et al.^3^	Male/30	Hypertension	Unknown	No	L	Calf and tight claudication progressive over four months
Rehman et al.^4^	Male/39	Dacron patch repair of the AFC 4 years earlier	Smoking	No	R	Claudication
Patel et al.^5^	Female/54	-	Ex-smoker	No	R	Acute right limb ischemia
Keiji et al.^6^	Male/53	-	Smoking	No	L	Hip and calf claudication
Esposito et al.^7^	Male/71	Hypertension, Hyperlipidaemia, Diabetes	Unknown	No	R	Intermittent claudication for 30 days
Kim et al.^8^	Male/59	Diabetes	Unknown	No	L	Swelling of the lower extremity
Steffen et al.^9^	Male/44	-	Ex-smoker	No	R	Nine month history of right thigh and calf claudication
Jindal et al.^10^	Female/53	-	-	Unknown	R	Claudication of the right thigh for six weeks
Jindal et al.^10^	Male/34	-	Smoking	Yes	R	Claudication of the right leg for one month
Kim et al.^11^	Male/56	Hyperlipidaemia	Smoking	No	R	Claudication for one month
Kim et al.^12^	Male/18	-	No	No	R	Right palpable inguinal mass, claudication for six months
Wu et al.^13^	Male/53	Hypertension	No	No	L	Intermittent claudication for three months, pulsating mass in his right groin
Lovelock et al.^14^	Female/37	-	No	No	L	Progressive claudication for 3–4 years, left calf pain
Dharmaraj et al.^15^	Female/52	-	No	No	L	Left sided claudication for 6 months

**Table 2:** Patient demographics, imaging modalities, affected arteries, overview.

***Demographics***	
Mean age (range), years	49 (18–79)
Men/women	12/5 (71%)
Right/Left/Both	9/8 (53%)/(47%)
Trauma	1 (6%)
Smoking	4 (24%)
***Symptoms***	**No. (%)**
Claudication	15 (88%)
Pulsating groin mass	3 (18%)
Limb swelling	1 (6%)
Acute ischemia	1 (6%)
***Imaging modality***	***No.(%)***
Duplex ultrasound	8 (47)
CTA	13 (76)
MRI/MRA	4 (24)
Catheter angiography	3 (18)
***Affected artery***	***No. (%)***
Common femoral	15 (88)
Superficial femoral	3 (18)
Profunda femoris	3 (18)
External iliac	3 (18)
Common femoral vein	1 (6)

**Table 3:** Pre-operative work-up.

	Ankle brachial index	Imaging modality	Correct pre-operative diagnosis (or suspicion) of CAD	Involved artery
Maeda et al.^1^	0.6	Duplex/CTA	Yes	Common femoral artery
Park et al.^2^	0.75	CTA	Yes	Common femoral artery
Gagnon et al.^3^	1.0	Duplex/CTA	Yes	Common femoral artery, deep femoral artery
Rehman et al.^4^	Unknown	Duplex/MRA	Yes	Common femoral artery, superficial femoral artery
Patel et al.^5^	unknown	Duplex/CTA	No	Common femoral artery
Keiji et al.^6^	0.80	CTA	No	Common femoral artery
Esposito et al.^7^	0.60	Duplex/CTA	No	Common femoral artery
Kim et al.^8^	-	CTA/MRA	Yes	Common femoral artery, common femoral vein
Steffen et al.^9^	-	Angiography	No	Common femoral artery, deep and superficial femoral artery
Jindal et al.^10^	-	Angiography	No	Common femoral artery
Jindal et al.^10^	-	Angiography	Yes	Common femoral artery
Kim et al.^11^	0.86	CTA	Yes	External iliac artery
Kim et al.^12^	0.57	Duplex/CTA/MRA	Yes	Common femoral artery
Wu et al.^13^	-	Duplex/CTA	No	Common femoral artery
Lovelock et al.^14^	-	CTA	No	External iliac artery, Common femoral artery
Dharmaraj et al.^15^	-	CTA	Yes	External iliac artery

**Table 4:** Treatment, joint connection, histological examination and follow up.

	Treatment	Joint connection	Histology	Follow up and outcome
Maeda et al.^1^	Cyst resection, autologous graft interposition (great saphenous vein)	No	Yes	Asymptomatic at three months
Park et al.^2^	Cyst resection with primary anastomosis	No	Yes	Recurrence after 200 days. Treated by cyst resection, autologous graft interposition (great saphenous vein) with, no recurrence at 12 months
Gagnon et al.^3^	Cyst resection, autologous graft interposition (great saphenous vein)	No	Yes	Asymptomatic at 12 months
Rehman et al.^4^	Cyst resection, patch repair	No	Yes	Recurrence after four years with replacement of a prosthetic graft bypass. No recurrence at six months
Patel et al.^5^	Cyst resection, autologous graft interposition (great saphenous vein)	No	Yes	Recurrence after two months treated by cyst resection, prosthetic graft interposition. No recurrence at six months follow up
Keiji et al.^6^	Cyst incision and decompression	No	Yes .	Recurrence after 20 days. Treated with cyst resection, prosthetic graft interposition. No recurrence at two years follow up
Esposito et al.^7^	Cyst resection, patch repair	No	Yes	Asymptomatic at 12 months
Kim et al.^8^	Cyst resection, patch repair	No	Yes	Unknown
Steffen et al.^9^	Cyst resection, patch repair	No	Yes	Asymptomatic at three months
Jindal et al.^10^	Cyst resection, autologous graft interposition (great saphenous vein)	No	Yes	Asymptomatic at four years
Jindal et al.^10^	Cyst resection, patch repair	No	Yes	Asymptomatic at one year
Kim et al.^11^	Cyst resection, prosthetic graft interposition, ligation of fistula	Yes	Yes	Asymptomatic at one year
Kim et al.^12^	Cyst resection, prosthetic graft interposition, ligation of fistula	Yes	Yes	Unknown
Wu et al.^13^	Cyst resection	No	Yes	Asymptomatic at six months
Lovelock et al.^14^	Cyst resection, autologous graft bypass (great saphenous vein)	No	Yes	Asymptomatic at six weeks
Dharmaraj et al.^15^	Cyst resection, Prosthetic graft bypass	No	Yes	-

**Table 5:** Treatment and reported recurrence, overview.

Procedure	No. (%)	Recurrence
Cyst resection	2 (12)	1 (treated with autologous vein reconstruction after recurrence without symptoms at 12 months)
Cyst resection with patch repair	5 (29)	1 (treated with prosthetic graft reconstruction without recurrence at six months)
Cyst resection with autologous vein reconstruction	6 (35)	1 (treated with prosthetic graft reconstruction without recurrence at six months)
Cyst resection with synthetic graft reconstruction	3 (18)	0
Cyst incision and decompression	1 (6)	1 (treated with prosthetic graft reconstruction without recurrence at 24 months)

## Discussion

Until now, there has been no clarity about the precise aetiology of CAD. Flanigan et al.^16^ drafted four theories of the possible aetiology of CAD. Those theories are referred to as the systemic disorder theory, traumatic theory, ganglion theory, and developmental theory.

The systematic disorder theory assumes that there is a systematic disorder of the connective tissue. The traumatic theory assumes that repetitive trauma causes possible destruction and cystic degeneration of the adventitia of the adjacent vessel. The ganglion theory suggests that joint related structures may undergo repeated trauma, with joint capsule degeneration resulting in connective tissue changes in which cells secrete collagen that contains hydroxyproline. These cells then form cysts around the joint capsule that may also invade the adventitia by creating a fistula and affect the arterial wall lumen. The developmental theory maintains that a joint related ganglion like structure is incorporated into the vessel during embryologic development. Desy and Spinner^17^ wrote a systematic review about CAD in all the arteries and veins. CAD was most seen in the popliteal artery (80.5%). In 122 cases (17%), a joint connection was identified on imaging and/or surgery. Most of them were found on CT or magnetic resonance imaging (MRI). A direct joint connection with the cyst was seen in all cases that had a pre-operative arthrogram (four cases). CTA gives the ability to assess the adventitia and the extension of cystic abnormalities outside the arterial lumen, and therefore giving more information of CAD pathology than angiography. Angiography is the modality mostly used in older articles because of the non-availability of CTA and therefore a reason to exclude articles before 1995. It is believed that a joint connection could easily be missed during surgery. This article strengthens the idea of the ganglion theory. The choice of a synthetic or venous graft depends on the extent of the CAD, the availability of a suitable venous graft, surgeon's preference, and patient's age. Therefore, a good pre-operative work up with CTA and additional MRI is recommended when suspecting CAD. If a connection to the joint is seen or identified, ligation is recommended to reduce the chance of recurrence. This could certainly be important to prevent recurrence when using an autologous graft, where the graft is less resistant to external forces in case of recurrence compared with a synthetic graft. Cyst resection or decompression alone may result in recurrence and the need for re-intervention, this is also reported in the treatment of CAD in other artery territories [17]. The pathogenesis of CAD is not fully understood, but joint connections are important in their development and treatment as described in this patient. The condition should be considered as a possible diagnosis when relatively young patients with few or no risk factors are seen with vascular symptoms.

### Conclusion

CAD generally affects middle aged men with a few or no risk factors for vascular disease. Cystic adventitial disease of the groin occurs unilateral in all reported cases. Patients mostly complain of claudication. In severe cases, it can lead to acute limb ischaemia. Limb swelling was described in one case due to a combined arterial and femoral vein involvement of CAD. Cyst resection with interposition of an autologous vein or synthetic graft seems to be the preferred treatment for CAD of the (ilio) femoral artery in larger affected sections, with a low reported recurrence rate. In local affected arteries, cyst resection with patch repair is also an option.

If a connection to the joint is seen or identified, ligation is recommended to reduce the chance of recurrence. CTA and MRI should be the imaging modality of choice when suspecting CAD to be able to make a more precise evaluation of the extension of the disease and subsequent narrowing of the lumen, enhanced soft tissue definition and identify joint connections to be able to make an appropriate operative plan. Joint connections could be important in the development and treatment of CAD to prevent recurrence.

## Conflict of interest

None.

## Funding

None.
